# Neural substrates of species-dependent visual processing of faces: use of morphed faces

**DOI:** 10.14814/phy2.12387

**Published:** 2015-05-13

**Authors:** Emi Yamada, Katsuya Ogata, Junji Kishimoto, Mutsuhide Tanaka, Tomokazu Urakawa, Takao Yamasaki, Shozo Tobimatsu

**Affiliations:** 1 Department of Clinical Neurophysiology, Neurological Institute, Graduate School of Medical Sciences, Kyushu UniversityHigashi-ku, Fukuoka, Japan; 2 Center for Clinical and Translational Research, Kyushu University HospitalHigashi-ku, Fukuoka, Japan

**Keywords:** Categorization, event-related potentials, face perception, inversion effect, morphing faces

## Abstract

Face identification and categorization are essential for social communication. The N170 event-related potential (ERP) is considered to be a biomarker of face perception. To elucidate the neural basis of species-dependent face processing, we recorded 128-ch high-density ERPs in 14 healthy adults while they viewed the images of morphed faces. The morphed stimuli contained different proportions of human and monkey faces, and the species boundary was shifted away from the center of the morph continuum. Three experiments were performed to determine how task requirement, facial orientation, and spatial frequency (SF) of visual stimuli affected ERPs. In an equal SF condition, the latency, and amplitude of the occipital P100 for upright faces were modulated in a monotonic-like fashion by the level of morphing. In contrast, the N170 latency for upright faces was modulated in a step-like fashion, showing a flexion point that may reflect species discrimination. Although N170 amplitudes for upright faces were not modulated by morph level, they were modulated in a monotonic-like fashion by inverted faces. The late positive (LP) component (350–550 msec) in the parietal region was modulated in a U-shaped function by morph level during a categorization task, but not in a simple reaction task. These results suggest that P100 reflects changes in the physical properties of faces and that N170 is involved in own-species selectivity. The LP component seems to represent species categorization that occurs 350 msec after stimulus onset.

## Introduction

Facial identification and categorization are fundamental for daily communication in primates. Adult primates process faces using a mature visual system that has years of experience with conspecific faces. Thus, adult humans and nonhuman primates exhibit superior facial recognition abilities within their own species (see review, Scott and Fava 2013). Electrophysiological studies have shown that the N170 event-related potential (ERP) is a well-known signature of the encoding stage of face structure (Bentin et al. 1996; Rossion et al. 1999a; Eimer 2000b). When people view monkey faces, N170 responses have longer latencies and larger amplitudes than when viewing other human faces (de Haan et al. 2002; Itier et al. 2011), although some studies have reported comparable amplitudes when viewing either monkey or human faces (Carmel and Bentin 2002; Itier et al. 2006). Moreover, viewing inverted faces produces larger N170 amplitudes and delayed latencies (Rossion et al. 1999a), an effect that is stronger for human faces than for animal faces (de Haan et al. 2002; Rousselet et al. 2004; Itier et al. 2006, 2011). At present, the N170 ERP is thought to be a neural marker for discriminating own from other species during face processing.

However, because most studies comparing human and animal faces have adopted stimuli with a clear species-boundary, modulation of the N170 ERP has not been fully elucidated in cases in which the species-boundary is unclear. Therefore, N170 characteristics in response to face stimuli that resemble both humans and monkeys (borderline monkey/human faces) remain unclear. Interestingly, Sigala et al. (2011) studied the activity of face-selective neurons in the inferior temporal (IT) cortex of monkeys when they viewed morphed faces on a continuum between human and monkey. They observed three main types of neuronal activity: (1) “Step-like” neurons, (2) “Inverted U-shaped tuning” neurons, and (3) “Monotonic-like” neurons. The “step-like” and “inverted U-shaped tuning” neurons were suggested to reflect neural processing of categorization (e.g., species boundary), while the activity of monotonic-like neurons reflected changes in the physical properties of the face stimuli (e.g., general preferences). A magnetoencephalographic study (Linkenkaer-Hansen et al. 1998) suggested that the N170 component is generated in fusiform and IT cortices. Additionally, categorizing faces by race produced an “other-race effect” on the N170 component (Balas and Nelson 2010; Senholzi and Ito 2013), suggesting that N170 might be related to face categorization. Therefore, if N170 is directly linked to face and species categorization, then modulation of N170 amplitude and latency along the morph level would show step-like or inverted U-shaped curves.

Although the P100 and N170 components are known to respond more strongly to faces than to objects (Eimer 1998; Goffaux et al. 2003; Itier and Taylor 2004), they can be affected by low-level features, such as contrast, luminance, and spatial frequency (SF) (Rossion and Jacques 2008). SF is especially relevant to measuring ERPs because increasing the SF of a stimulus can diminish the perceived difference between faces and objects (Goffaux et al. 2003). However, faces have been found to produce much larger ERP amplitudes than houses under an equated SF condition (Rousselet et al. 2005). Thus, we considered that it was necessary to compare the controlled and uncontrolled SF conditions to clarify the difference in actual ERP responses between human and monkey faces.

Face identification and emotional analysis can also modulate ERP components of face processing. Using a face/car categorization task, an ERP study has indicated that late positive (LP) components (350–450 msec) might reflect decision-making processes and behavioral judgment (Philiastides and Sajda 2006). Therefore, to clearly determine the mechanisms related only to the categorization process, we separated it from the recognition processes using a categorization task and a simple reaction task. Thus, only if P100, N170, or LP characteristics differ between tasks, can we say that that particular component is involved in the categorization process.

Our aims were to (1) investigate how ERP components are modulated by morphed human-monkey faces when SF is controlled and to (2) distinguish species-sensitive ERP modulation from categorization processes.

## Material and Methods

We conducted three experiments on six different days (each experiment taking 2 days to complete) to determine the effects of three factors on major ERP components: face orientation (upright vs. inverted), SF (equal vs. unequal), and task requirement (categorized vs. uncategorized). Each experiment was composed of a different combination of the three factors. For example, Day 1 used upright faces, equal SF, and a categorization task, while Day 2 used inverted faces, equal SF, and a categorization task. All experiments were performed with the same healthy subjects.

### Experiments 1 and 2: ERPs during the categorization task

Two experiments were conducted using a categorization task to determine the effect SF and orientation of the morphed faces had on ERPs. Experiment 1 used equal SFs, while Experiment 2 did not. Although the two experiments only differed with respect to SF, we dealt with them separately because we compared the effect of task requirement on equal SF stimuli (see section Experiment 3: ERPs in a simple reaction task).

#### Participants

Fourteen right-handed healthy Japanese adults (six females, mean age 23.6 years, ranging from 20 to 31 years) were recruited to participate in Experiments 1 and 2. Both experiments were performed on different days under two conditions: upright or inverted facial orientation. The interval between the two experiments ranged from 2 days to several months because of the participants’ schedules. All participants were undergraduate or graduate students at Kyushu University. We confirmed that all participants were naive regarding monkey-face stimuli and had normal or corrected-to-normal vision. No participants had a history of neurological or psychiatric disorders. In accordance with The Code of Ethics of the World Medical Association (Declaration of Helsinki), informed consent was obtained from each participant after the nature of the experiments had been fully explained. The experimental procedures were approved by the Ethics Committee at the Graduate School of Medical Sciences, Kyushu University.

#### Visual stimuli

**Figure 1:**
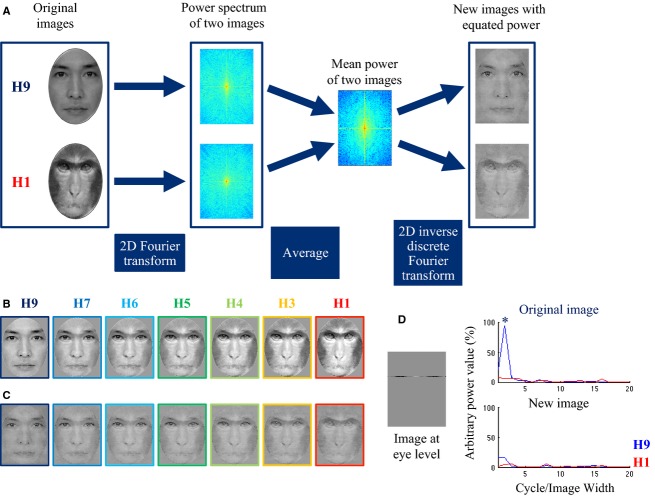
(A) Method for equating spatial frequency (SF) in terms of power spectra of the images in morphed faces containing varying proportions of human and monkey faces. We used MATLAB to adjust the SF, mean luminance, and contrast. Each category represents the human to monkey-face ratio (e.g., H9 contains 90% human face). (B) Morphed faces with uncontrolled SF information. (C) Morphed faces with equated SF information. (D) Power spectra of SF (cycles/image width) at the eye level of H9 (blue line) and H1 (red line) were obtained by one dimensional Fast Fourier Transform. At low SFs, power values for the original H9 image were higher than those for the H1 image (asterisk), but this peak virtually disappeared after image processing.

The morphed faces were created by combining human and monkey faces (Fig.1). We used Morpher 3.1 (Rotshtein et al. 2005) to generate a continuum of 11 images, in which the human ratio of each stimulus was reduced in 10% steps (from 100% to 0%). We confirmed the change in linearity using a pixel-based comparison that computed the root mean squared error (RMSE) between pairs of morphing faces (Sigala et al. 2011). RMSE is useful for quantifying differences between pairs of images in terms of their global contrast and luminance levels. RMSEs for eight pairs of images (H9 vs. H8, H8 vs. H7, H7 vs. H6, H6 vs. H5, H5 vs. H4, H4 vs. H3, H3 vs. H2, and H2 vs. H1) were computed and the mean RMSE was 6.60 ± 0.02. This indicates that images from adjacent morph levels differed by a similar amount in terms of basic image properties. From this it follows that the morphs themselves do not contain any clear boundaries or discontinuities, which is consistent with being generated by linear interpolation.

The original human face was a Japanese male face with neutral expression from the ATR face database (ATR Promotions, Inc., Kyoto, Japan) and the original monkey face was a Japanese macaque. We carried out a psychological categorization task (*n* = 10; four females; mean age, 29.5 years; range, 21–42 years) using nine morphed faces (human/monkey [H/M] ratios: 9/1 [H9] 8/2 [H8] 7/3 [H7], 6/4 [H6], 5/5 [H5], 4/6 [H4], 3/7 [H3], 2/8 [H2], and 1/9 [H1]) to choose faces that would be suitable for ERP experiments. None of the subjects in this psychological experiment participated in the following EEG experiments. The response accuracy (human or monkey, each with a probability of 0.5) of H8 (2/8) and H2 (8/2) was approximately 100%, similar to H9 (9/1) and H1 (1/9) combinations. Therefore, we did not use these combinations, but selected the remaining seven: H9, H7, H6, H5, H4, H3, and H1 (Fig.1B). These seven images were cropped with an oval window to remove external features. Then we created a set of stimuli with equal SF (cycles/image width) in terms of image power spectrum. First, the morphed faces were decomposed into Fourier power spectra using a 2D fast Fourier transformation. Then, the mean SF power was calculated and transformed to each corresponding image using a 2D inverse discrete Fourier transform procedure (Fig.1A and D). This enabled us to make broadband SF stimuli that included both low and high SF. The images were resized to 300 × 385 pixels, which subtended a visual angle of 5.3 × 6.8° at a viewing distance of 114 cm. We made two sets of morphed faces: one set equated to mean luminance (36 cd/m^2^), mean contrast, and SF content (Fig.1C), while the other equated to mean luminance (36 cd/m^2^) and mean contrast (Fig.1B). After morphing, all image processing was conducted using MATLAB ver. 7.4 (The MathWorks Inc., Natick, MA).

#### Experimental procedures

Participants were seated comfortably in a semi-darkened room and instructed to attend a fixation point at the center of a cathode-ray-tube monitor with a resolution of 1024 × 768 pixels. We used Presentation software (Neurobehavioral Systems, Albany, CA) to display the morphed faces in either upright or inverted orientations. Visual stimuli were presented for 500 msec on a gray background with a mean luminance that was equal to that of the stimuli. Inter-stimulus intervals were randomized between 1500 and 2500 msec. Each type of morphed face was presented 144 times in a pseudorandom order. Thus, the total number of stimuli in one experiment was 1008 (144 × 7). Participants were instructed to press a button labeled “human” as quickly as possible if the image more closely resembled a human or to press a second button if the image more closely resembled a monkey. If subjects did not press either button before the next stimulus appeared, the trial was regarded as invalid and excluded from analysis. Half of the participants were instructed to press the button with the right index finger while the other half were asked to use the left. From the behavioral data, we were able to calculate the accuracy of judgment and the mean reaction time (RT) to the faces.

#### ERP recording and data analysis

We used a high-density 128-channel system (NetAmps 200, Electrical Geodesics Inc., Eugene, OR) to record ERPs. ERP data were obtained with a vertex electrode (Cz) as a reference. The data were band-pass filtered between 0.01 and 400 Hz and digitized at a sampling rate of 1000 Hz. ERPs were processed offline using Net Station 4.2 software (Electrical Geodesics). The data were filtered using a 0.5–30 Hz bandpass filter and segmented from 100 msec before and 600 msec after the stimulus onset. Trials were rejected if the amplitude exceeded 100 *μ*V or if they contained more than 10 bad channels due to eye movements in excess of 55 *μ*V. In the remaining trials, data for bad channels were interpolated from the remaining channels. Data were then re-referenced to the average of the two electrodes closest to the tip of the nose (Horie et al. 2012). We conducted at least 100 trials for each participant in each condition, and corrected the baselines using the interval from 100 to 0 msec before stimulus onset.

To determine the appropriate time windows for major ERP components, a topographic consistency test (TCT) was applied to the data. TCT is a method that uses randomization techniques to reveal the times at which the topographies are consistent across participants or trials. For accurate statistical analysis, TCT should use the false discovery rate (FDR) or multiple comparisons (Koenig and Melie-Garcia 2010; Tzovara et al. 2012). In this study, we adopted the use of the FDR. Furthermore, we applied TCT to difference waveforms, as has been done elsewhere (Stefanics and Czigler 2012).The components and time windows were estimated using the waveforms produced by subtracting H1 waveforms from those of H3 to H9. We found two major early components (P100 and N170) and one LP component using this method, and evaluated the properties of each. Amplitudes were measured from baseline. The region of interest (ROI) for each component was defined as the electrode that recorded the maximum response and its three adjacent electrodes. We automatically computed a peak electrode for each time window using MATLAB, and then the ROI electrodes were selected by visual inspection based on each individual's scalp topography. P100 latencies and amplitudes were measured at a positive peak between 90 and 130 msec around electrodes at O1/O2 (International 10–20 system). N170 latencies and amplitudes were measured from the negative peak between 130 and 200 msec around electrodes at T5/T6. The LP amplitudes were averaged between 350 and 550 msec.

### Experiment 3: ERPs in a simple reaction task

Experiment 3 adopted a simple reaction task in which faces did not need to be identified or classified. The procedure was the same as for Experiment 1. By comparing the ERP responses from this task with those obtained from Experiment 1, we were able to isolate neural mechanisms related to the categorization process.

### Materials and methods

Twelve participants from Experiments 1 and 2 participated in Experiment 3 (five females, mean age 23.2 years, ranging from 20 to 31 years). We used the set of SF controlled stimuli (Fig.1C). Experimental protocol, ERP recoding, and data analysis were the same as in Experiments 1 and 2; however, task requirement was different. Participants pressed the button simply to indicate that a stimulus had appeared.

### Statistical analyses

Although we performed the three experiments separately, Experiments 1 and 2 were treated as a single experiment with a 2 (SF: equal, unequal) × 2 (orientation: upright, inverted) × 7 (morph type) × 2 (laterality) design to directly assess the effects of SF on the ERP components. Similarly, Experiments 1 and 3 were combined into a single dataset to directly assess the effects of task requirement on ERP components. Thus, all data were subjected to a repeated measures analysis of variance (ANOVA) with four factors (orientation, morph type, laterality, and either SF or task requirement). Furthermore, when necessary, a three-way ANOVA or a two-way ANOVA was used to determine the effect of each factor on the ERP components and behavioral data.

If the four-way ANOVA revealed a significant main effect of morph type or a significant interaction between morph type and any of the other factors, we evaluated how morph type modulated the ERP components. To characterize the relationship between the seven morph types (Fig.1B and C) and the ERP components, we adopted either a linear regression model or a second-order polynomial regression model. We assessed the fitness of our model using a lack-of-fit (LOF) test to determine whether the relationship was linear or nonlinear. If LOF is significant, it indicates that a linear regression model is not the optimal fit for the data (Andersen et al. 1990). If there was a significant main effect of morph type or an interaction, we defined one of three modulation-transfer functions as the human ratio. First, if the ERP components were fitted to a linear regression model without significant LOF, we considered the responses to have been modulated by a monotonic-like function (or a linear function). Second, if the ERP components were fitted to a linear regression model with significant LOF, we considered the responses to have been modulated by a step-like function. Third, if the ERP components were fitted to a second-order polynomial regression model, we considered the responses to have been modulated by a U-shaped or an inverted U-shaped function. Additionally, we computed Pearson's correlation coefficients between face type and P100 components and between face type and N170 components to characterize the linear or nonlinear relationship for P100 and N170. For the ERP tuning properties, we adopted the following functions: [0 0 0 0 1 1 1] for “step-like”, [0.1 0.3 0.4 0.5 0.6 0.7 0.9] for “monotonic like”, and [1 0.5 0 0 0 0.5 1] for “U-shaped”. If we found significant correlations between face type and either component, it was labeled as one of the three function types. If we found more than one significant correlation, we chose the function type that had the larger *P*-value.

All statistical analyses were performed using the JMP statistical software package (SAS Institute Inc., Cary, NC) and *P *<* *0.05 was regarded as significant. Multiple comparison post hoc analysis was conducted using Tukey's HSD.

## Results

### Behavioral data

#### Behavioral data from Experiments 1 and 2: the effect of categorization

**Figure 2:**
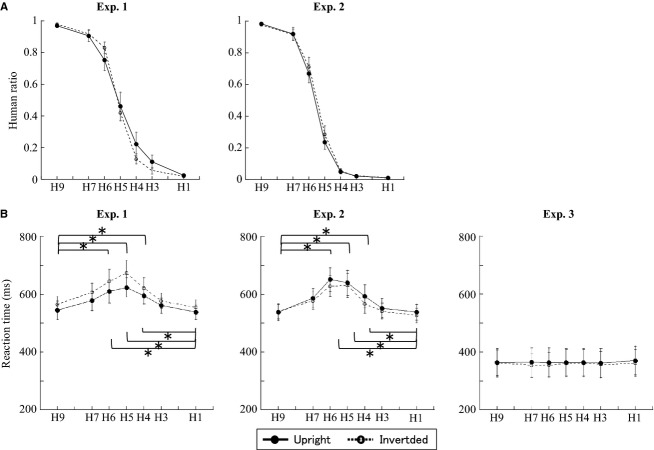
Behavioral results from all three experiments. (A) Judgment of a stimulus as mostly human decreased gradually as a function of the human ratio in Experiments 1 and 2. (B) Reaction times (RTs) in the categorization task (Experiments 1 and 2) fit an inverted U-shaped curve, but were unaffected by morph level in the simple reaction task (Experiment 3). No behavioral results were significantly changed by face orientation. Solid lines with filled circles, upright faces; dashed lines with unfilled circles, inverted faces. Error bars indicate SEM in this and Figures7 *P <* *0.05.

The probability of judging a face as human decreased with the human-face proportion of the morphed faces (Fig.2A). Results from a three-way ANOVA (SF × orientation × morph type) of the combined behavioral data of Experiments 1 and 2 are summarized in Table1. A significant main effect was found for morph type, however, no significant interactions between morph type and SF or morph type and orientation were found.

**Table 1:** Three-way ANOVA results for the RT data

	Factor	df	*F*	*P*
Combined Experiments 1 and 2				
RT	Spatial frequency	1	0.71	0.40
	Orientation	1	0.018	0.89
	Morph type	6	**22.2**	**<0.001**
	Morph type × spatial frequency	6	0.56	0.76
	Morph type × orientation	6	0.17	0.98
Combined Experiments 1 and 3				
RT	Task requirement	1	**52.3**	**<0.001**
	Orientation	1	0.016	0.90
	Morph type	6	1.90	0.08
	Morph type × task requirement	6	2.06	0.057
	Morph type × orientation	6	0.071	0.99

ANOVA, analysis of variance; RT, reaction time. Bold values indicate *P* < 0.05.

Similarly, a three-way ANOVA revealed a significant main effect of morph type on RT, and no significant interaction between SF and face orientation. A post hoc analysis revealed significant differences between stimuli with ambiguous faces and those with less-ambiguous ones (i.e., stimuli at the ends of the continuum) (H9 and H1 vs. H7–H4) (Fig.2B). Thus, the RT data produced an inverted U-shaped curve, with a peak at an intermediate morph level.

#### Behavioral data from Experiments 1 and 3: the effect of simple reaction task

A three-way ANOVA only showed a significant main effect of task (Table1). To clarify the effects of morph type and orientation on the LP component in Experiment 3, we applied a two-way ANOVA (morph type and orientation) to the data. There were no significant differences in RTs across the face conditions (Fig.2B) and no significant interactions.

### ERP data

#### ERP data for Experiments 1 and 2: the effect of categorization

The TCT indicated three periods in which the greatest amounts of activity in the ERP signal occurred in individual ERPs. They corresponded to two typical early components (P100 and N170) and one late component (LP) between 350 and 550 msec after the stimulus onset. We used these time windows defined by TCT.

**Figure 3:**
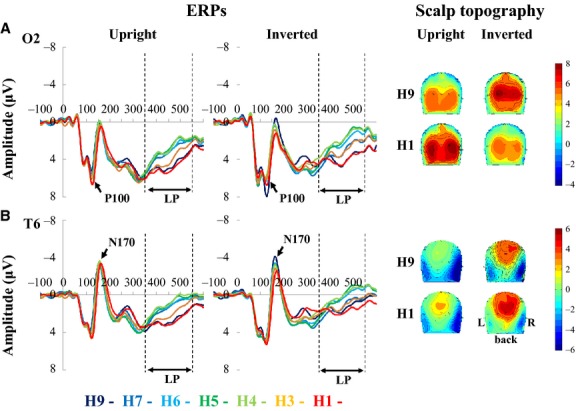
Grand averaged waveforms and scalp topography of ERPs at O2 (A) and T6 (B) in Experiment 1 (*n* = 14). Note that P100 at the occipital region and N170 at the occipitotemporal region were elicited as major early components. (A) P100 for inverted faces was larger than that for upright faces in the H9 and H1 conditions. (B) In contrast, N170 at T6 was clearly larger in response to H9 in the upright condition than to H1 in the inverted condition.

**Figure 4:**
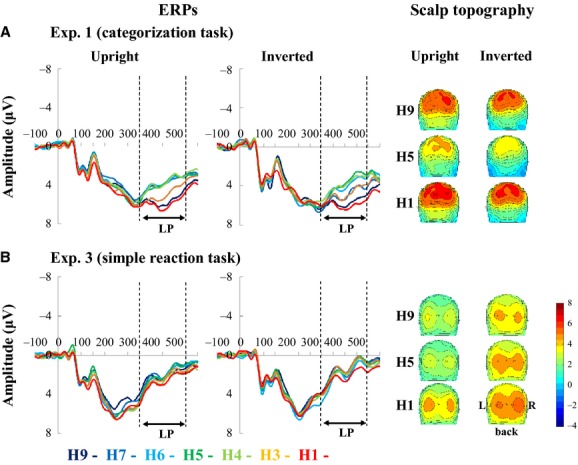
Grand averaged ERP waveforms and scalp topography at P4 in Experiments 1 and 3 (*n* = 12). Note that the LP amplitudes at P4 in the H9 and H1 conditions are larger than those in the H5 condition in Experiment 1 (A), while the LP amplitudes in Experiment 3 are not well defined at P4; moreover, they do not differ among the stimuli (B). ERP, event-related potential; LP, late positive.

Figures3, 4 show the grand-averaged ERPs from Experiment 1. We found the two major early components and one LP component for all stimuli. P100 was predominantly recorded in the occipital area (O1 and O2) (Fig.3A). Six participants showed two peaks at around 100 msec, so we evaluated the scalp topography of each participant and determined that a P100 peak was genuine if both occipital areas were activated. N170 was predominantly distributed over the occipitotemporal region (T5 and T6) (Fig.3B) and lateralized to the right side. Scalp topographies revealed LP was produced over the parietal regions. Thus, the electrodes around P3/P4 (Fig.4A) were chosen for further analysis.

#### Effects of SF, orientation, morph type, and laterality on ERP components

Four-way ANOVA results of the combined ERP data in Experiments 1 and 2 are summarized in Table2. We found a significant main effect of SF on P100, N170, and LP. A significant main effect of face orientation was also observed on P100 and N170 latencies and LP amplitude, while a main effect of laterality was found on P100 and N170 latencies. Additionally, we found significant effects of morph type on N170 latency and LP amplitude. A significant main effect of laterality was evident on P100 latency but not on P100 or LP amplitudes. A significant effect of laterality was also apparent for the N170 component (Table2, Fig.3A).

**Table 2:** Four-way ANOVA for the effects of spatial frequency, orientation, morph type, and laterality on the amplitudes and latencies of the P100, N170, and LP components

Factor	Latency			Amplitude		
	df	*F*	*P*	df	*F*	*P*
P100						
Spatial frequency	1	**50.7**	**<0.001**	1	**5.28**	**0.022**
Orientation	1	**14.7**	**<0.001**	1	0.88	0.35
Morph type	6	0.62	0.71	6	1.67	0.12
Laterality	1	**5.26**	**0.022**	1	0.22	0.64
Morph type × spatial frequency	6	1.53	0.17	6	0.89	0.50
Morph type × orientation	6	0.70	0.65	6	**5.73**	**<0.001**
Morph type × laterality	6	0.17	0.98	6	0.57	0.75
N170						
Spatial frequency	1	**71.3**	**<0.001**	1	**12.9**	**<0.001**
Orientation	1	**51.2**	**<0.001**	1	0.00	0.99
Morph type	6	**4.79**	**<0.001**	6	2.01	0.062
Laterality	1	**4.58**	**0.033**	1	**4.60**	**0.032**
Morph type × spatial frequency	6	**2.32**	**0.032**	6	0.30	0.94
Morph type × orientation	6	0.78	0.58	6	**2.72**	**0.013**
Morph type × laterality	6	0.44	0.85	6	0.25	0.96
LP						
Spatial frequency				1	**4.82**	**0.028**
Orientation				1	**6.42**	**0.012**
Morph type				6	**20.7**	**<0.001**
Laterality				1	0.001	0.97
Morph type × spatial frequency				6	1.13	0.34
Morph type × orientation				6	1.44	0.20
Morph type × laterality				6	0.203	0.98

Note that data from Experiments 1 and 2 (*n* = 14) were combined. ANOVA, analysis of variance; LP, late positive. Bold values indicate *P* < 0.05.

**Figure 5:**
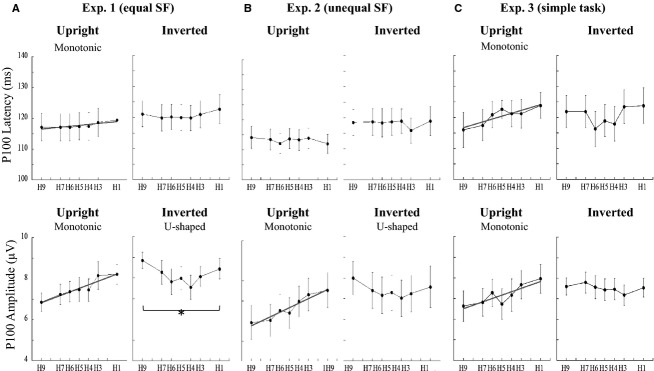
Changes in mean P100 latencies and amplitudes at the electrodes around O2 in the three experiments. (Top Row) P100 latencies for upright faces are modulated in a monotonic-like fashion by morph type in Experiments 1 and 3 (A and C), but not in Experiment 2 (B). (Bottom Row) In both experiments, P100 amplitudes for upright faces significantly increase monotonically as faces appear less human (A–C). However, P100 amplitudes for inverted faces showed a different trend (U-shaped function). The solid line represents the significant regression line.

For P100 amplitude, a four-way ANOVA showed a significant interaction between face orientation and morph type (Table2). P100 amplitude for upright faces was modulated in a monotonic fashion similar to P100 latency with a significant main effect of morph type by a linear regression model (Table3, Fig.5A and B). However, in contrast to upright faces, P100 amplitude for inverted faces was modulated by a U-shaped function as indicated by the fit to a nonlinear regression model (Table3, Fig.5A). Thus, we used a two-way ANOVA (morph type × laterality) to clarify the effect of morph type. A significant main effect of morph type (*F*_(6, 169)_ = 2.64, *P *=* *0.02) was found in Experiment 1. Interestingly, while P100 latencies in Experiment 2 were not affected by morph type (upright: *P *=* *0.93, inverted: *P *=* *0.27, Fig.5B), amplitude modulation in Experiment 2 was similar to that in Experiment 1 (upright: *F*_(6, 169)_ = 8.63, *P *<* *0.001, inverted: *F*_(6, 169)_ = 1.49, *P *=* *0.18, Fig.5B).

**Table 3:** The relationship between morph type and P100 components obtained by the three regression models and Pearson correlation

Regression model	Orientation	Function	Regression model (*R*^2^, *P*)	LOF (*P*)
Exp. 1 (equal SF)				
Latency	Upright	Monotonic	Linear (0.74, *P* = 0.01)	0.34
	Inverted	–	–	–
Amplitude	Upright	Monotonic	Linear (0.90, *P *=* *0.001)	0.80
	Inverted	U-shaped	2 order Polynomial (0.86, *P *=* *0.009)	0.86
Exp. 2 (unequal SF)				
Latency	Upright	–	–	–
	Inverted	–	–	–
Amplitude	Upright	Monotonic	Linear (0.92, *P *<* *0.001)	0.43
	Inverted	U-shaped	2 order Polynomial (0.93, *P *=* *0.002)	0.14
Exp. 3 (simple task)				
Latency	Upright	Monotonic	Linear (0.79, *P *=* *0.007)	0.83
	Inverted	–	–	–
Amplitude	Upright	Monotonic	Linear (0.76, *P *=* *0.01)	0.99
	Inverted	–	–	–

Pearson correlation	Orientation	Function	Monotonic	Step-like	U-shaped
			(0.1 0.3 0.4 0.5 0.6 0.7 0.9)	(0 000 1 11)	(1 0.5 0 00 0.5 1)
Exp. 1 (equal SF)					
Latency	Upright	Monotonic	***P *****=***** *****0.007**	***P *****=***** *****0.03**	*P *=* *0.29
	Inverted	–	–	–	–
Amplitude	Upright	Monotonic	***P *****<***** *****0.001**	***P *****=***** *****0.03**	*P *=* *0.66
	Inverted	U-shaped	*P *=* *0.44	*P *=* *0.56	***P *****=***** *****0.03**
Exp. 2 (unequal SF)					
Latency	Upright	–	–	–	–
	Inverted	–	–	–	–
Amplitude	Upright	Monotonic	***P *****<***** *****0.001**	***P *****=***** *****0.002**	*P *=* *0.55
	Inverted	U-shaped	*P *=* *0.33	*P *=* *0.50	***P *****=***** *****0.02**
Exp. 3 (simple task)					
Latency	Upright	Monotonic	***P *****=***** *****0.003**	*P *=* *0.19	*P *=* *0.40
	Inverted	–	–	–	–
Amplitude	Upright	Monotonic	***P *****=***** *****0.005**	***P *****=***** *****0.02**	*P *=* *0.62
	Inverted	–	–	–	–

LOF, lack-of-fit; SF, spatial frequency. Bold values indicate *P* < 0.05.

**Figure 6:**
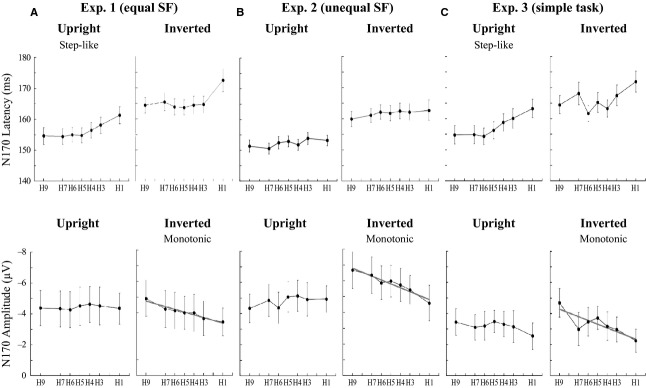
Changes in mean N170 latencies and amplitudes at the electrodes around T6 in the three experiments. (top row) In Experiments 1 and 3 (A and C), N170 latencies were significantly prolonged as faces appeared less human, although the data did not fit a linear regression model in either condition. These latencies were modulated by a step-like fashion. Although the latencies in Experiment 2 were not modulated in a step-like fashion (B), the N170 amplitude for upright faces was not significantly affected by morph type in either experiment (A–C). N170 amplitude for inverted faces decreased in all Experiments (monotonic-like fashion) as the faces appeared less human. The solid line represents the significant regression line of morph type. In terms of the data of individual subjects, eight of 14 subjects in Experiment 1; seven of 14 subjects in Experiment 2; and five of 12 subjects in Experiment 3 showed a linear relationship as seen in the group data.

For N170 latency, a four-way ANOVA showed a significant interaction between face orientation and morph type (Table2). Therefore, a three-way ANOVA was conducted and significant main effects were found for orientation (*F*_(6, 371)_ = 204, *P *<* *0.001), laterality (*F*_(6, 371)_ = 35.4, *P *<* *0.001), and morph type (*F*_(6, 371)_ = 21.6, *P *<* *0.001). We also applied a linear regression model and LOF test. The analysis revealed a significant main effect of morph type with a significant LOF: the mean latencies increased as the morphed faces appeared less human. However, the relationship between latency and morph type was fit best by a step-like function, rather than a linear one in Experiment 1 (Table4, Fig.6A). In Experiment 2, a linear regression model revealed main effects of laterality and morph type without significant LOF. Therefore, N170 latencies in Experiment 2 were modulated by a monotonic-like function (Table4, Fig.6B).

**Table 4:** The relationship between morph type and N170 components obtained by the three regression models and Pearson's correlation

Regression model	Orientation	Function	Regression model (*R*^2^, *P*)	LOF (*P*)
Exp. 1 (equal SF)				
Latency	Upright	Step-like	Linear (0.74, *P* = 0.008)	**0.009**
	Inverted	–	–	–
Amplitude	Upright	–	–	–
	Inverted	Monotonic	Linear (0.86, *P* < 0.001)	0.99
Exp. 2 (unequal SF)				
Latency	Upright	–	–	–
	Inverted	–	–	–
Amplitude	Upright	–	–	–
	Inverted	Monotonic	Linear (0.94, *P* < 0.001)	0.85
Exp. 3 (simple task)				
Latency	Upright	Step-like	Linear (0.83, *P* = 0.003)	**0.049**
	Inverted	–	–	–
Amplitude	Upright	–	–	–
	Inverted	Monotonic	Linear (0.71, *P* = 0.02)	0.45

Pearson's correlation	Orientation	Function	Monotonic	Step-like	U-shaped
			[0.1 0.3 0.4 0.5 0.6 0.7 0.9]	[0 0 0 0 1 1 1]	[1 1 0 0 0 1 1]
Exp. 1 (equal SF)					
Latency	Upright	Step-like	***P***** = 0.004**	***P***** = 0.003**	*P* = 0.42
	Inverted	–	–	–	–
Amplitude	Upright	–	–	–	–
	Inverted	Monotonic	***P***** < 0.001**	***P***** = 0.04**	*P* = 0.97
Exp. 2 (unequal SF)					
Latency	Upright	–	–	–	–
	Inverted	–	–	–	–
Amplitude	Upright	–	–	–	–
	Inverted	Monotonic	***P***** < 0.001**	***P***** = 0.03**	*P* = 0.84
Exp. 3 (simple task)					
Latency	Upright	Step-like	***P***** = 0.007**	***P***** = 0.002**	*P* = 0.52
	Inverted	–	–	–	–
Amplitude	Upright	–	–	–	–
	Inverted	Monotonic	***P***** = 0.01**	*P* = 0.10	*P* = 0.73

LOF, lack-of-fit; SF, spatial frequency. Bold values indicate *P* < 0.05.

While N170 amplitudes gradually decreased along the face continuum for inverted faces, this was not the case for upright faces (Fig.6A). Thus, an interaction between orientation and morph type was significant. The data were well fitted to a linear regression model, suggesting that N170 amplitudes were modulated in a monotonic fashion (Table4).

**Figure 7:**
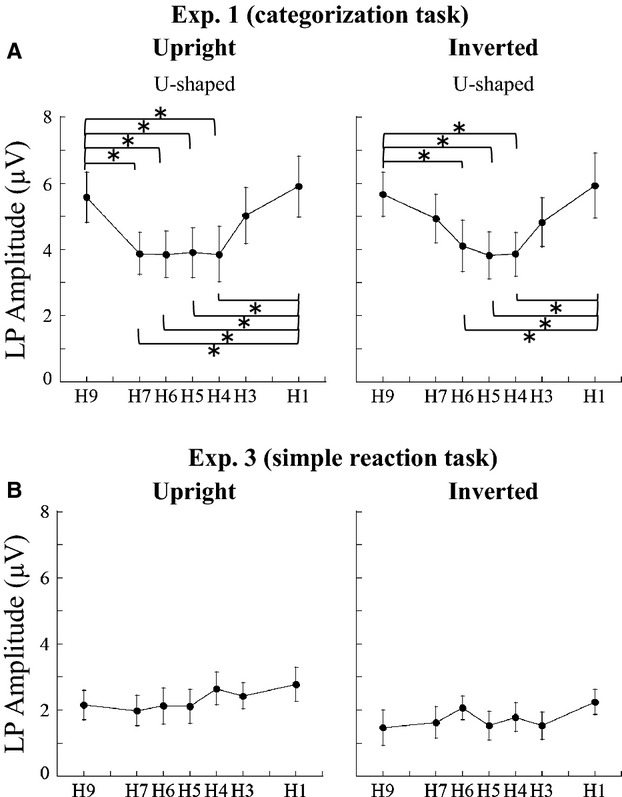
Changes in mean late positive (LP) amplitudes at the electrodes around P4 in Experiments 1 and 3. (A) The categorization task. LP amplitudes fit a U-shaped curve as a function of human-face proportion in Experiment 1. The LP amplitudes were significantly different between the middle and ends of the face continuum. *P <* *0.05. (B) The simple reaction task. Although a significant main effect of morph type was observed in the simple reaction task, post hoc analysis revealed that the H1–H9 conditions did not differ significantly.

Among the seven morphed faces, mean LP amplitudes were largest for the least morphed stimuli (H1 and H9), and formed U-shaped curves along the face continuum (Fig.7A). A post hoc analysis revealed significant differences between the middle and end points of upright and inverted continuums (H9 and H1 vs. H7–H4), regardless of laterality (Table2, Fig.7A).

#### Effects of task requirement, orientation, morph type, and laterality on ERP components

Results from a four-way ANOVA using combined ERP data from Experiments 1 and 3 are summarized in Table5. We found a significant main effect of task requirement on N170 and LP amplitudes, significant main effects of morph type on P100 and N170 latency, and N170 and LP amplitude (Table5), and a significant effect of laterality on N170 latency. No main effect of orientation was found for any component.

**Table 5:** Four-way ANOVA for the effects of task requirement, orientation, morph type, and laterality on the amplitudes and latencies of the P100, N170, and LP components

Factor	Latency			Amplitude		
	df	*F*	*P*	df	*F*	*P*
P100						
Task requirement	1	3.32	0.069	1	2.30	0.13
Orientation	1	0.89	0.34	1	1.11	0.29
Morph type	6	**2.20**	**0.041**	6	1.46	0.19
Laterality	1	0.89	0.34	1	0.060	0.81
Morph type × task requirement	6	0.14	0.99	6	0.74	0.62
Morph type × orientation	6	0.92	0.48	6	**3.26**	**0.004**
Morph type × laterality	6	0.35	0.91	6	0.62	0.72
N170						
Task requirement	1	2.34	0.13	1	**7.46**	**0.007**
Orientation	1	**62.2**	**<0.001**	1	0.16	0.69
Morph type	6	**22.3**	**<0.001**	6	**4.04**	**<0.001**
Laterality	1	**3.92**	**0.048**	1	0.75	0.38
Morph type × task requirement	6	0.63	0.71	6	0.28	0.94
Morph type × orientation	6	2.04	0.059	6	1.98	0.066
Morph type × laterality	6	0.48	0.83	6	0.021	1.00
LP						
Task requirement				1	**108**	**<0.001**
Orientation				1	1.35	0.25
Morph type				6	**7.75**	**<0.001**
Laterality				1	0.25	0.61
Morph type × task requirement				6	**5.43**	**<0.001**
Morph type × orientation				6	1.21	0.30
Morph type × laterality				6	0.082	0.99

Note that data from Experiments 1 and 3 (*n* = 12) were combined. ANOVA, analysis of variance; LP, late positive. Bold values indicate *P* < 0.05.

Although P100 latencies in Experiments 1 and 3 gradually increased as the human-face proportion decreased in the upright face condition (Table3, Fig.5A), post hoc analysis revealed no significant differences between morph types. The P100 latency for upright faces was fitted to a linear regression model without significant LOF, suggesting that P100 latency for upright faces was modulated in a monotonic-like fashion (Table3, Fig.5A and C).

The modulations of N170 latency and amplitude in Experiment 3 were similar to those in Experiment 1 (Table4, Fig.6A and C). Particularly, the modulation of N170 latencies for upright faces had a flexion point in both experiments.

LP scalp topography showed ill-defined activation in parietal regions (Fig.4B) in Experiment 3 that were almost of the same magnitude regardless of morph type (Fig.7B). Thus, we did not observe the U-shaped curve function found in Experiment 1 (Fig.7B).

## Discussion

In this study, we used morphed faces to investigate species-specific sensitivity for face processing. The effects of laterality (right hemisphere predominance) and orientation on ERP components were similar to results reported in other studies of face perception (Bentin et al. 1996). Three major findings are summarized as follows. First, our morphed stimuli included varying proportions of human and monkey faces with equal or unequal SFs. We found that SF significantly affected early ERP components, particularly N170 latency and amplitude. Thus, controlling SF was necessary for detecting face recognition related to species-specific modulation of P100 and N170. Second, we sought to elucidate the influence of categorization/classification processes on behavior and ERP components. Our results suggest that ERPs and RT were affected by whether or not participants were required to categorize the faces. Third, each ERP component was differently influenced by SF, orientation, and morph level. One may argue that the experimental conditions were not fully counterbalanced across the different days, which means that the main factors “SF” and “task requirement” are confounded with the order. However, the time between each experimental condition was long enough to reduce any practical effects or habituation. Therefore, we believe that our data were not significantly influenced by the order of experiments. In the following sections, we discuss species-specific sensitivity for face processing.

### P100 detects changes in the physical properties of face stimuli

Although a study has compared P100 amplitudes elicited by viewing human and monkey faces (Balas and Stevenson 2013), it reported no main effect of species. Here, we found that, for upright faces, P100 latency and amplitude were modulated in a monotonic-like fashion by the degree to which the faces appeared human. Additionally, P100 amplitude was larger for monkey faces than for human faces. These findings are unexpected because the P100 component has been hypothesized to reflect processing of low-level visual features (Rossion and Jacques 2008). The contribution of low-level visual features is unlikely to explain this phenomenon because SF, mean luminance, and mean contrast were controlled in Experiment 1. Additionally, manipulating the SF but not task requirement significantly altered the P100 amplitude (Experiments 2 and 3). Therefore, modulation of P100 amplitude likely resulted from activity related to facial processing. Furthermore, we were especially surprised that unlike N170, upright human faces elicited a smaller P100 than did the monkey face (Fig.5A–C). A face-priming study has shown that when the same set of faces is viewed twice, the second presentation elicits a larger P100 amplitude than does the first presentation (Herrmann et al. 2007). These authors stated that this enhanced P100 might result from an increase in attention toward the area in which the second visual stimulus was expected to appear. Another study using emotional face stimuli revealed increased responses to fearful faces in the primary visual cortex at ~100 msec, while inverted faces produced no such effect (Pourtois et al. 2004). These two phenomena – increased attention for repeated stimuli and increased response to fearful faces – were only found to affect the P100 component when the face stimuli were presented in an upright orientation. Additionally, faces that deviated from a prototypical face were shown to elicit larger P100 amplitudes (Halit et al. 2000), which could be explained in terms of attentional modulation based on a prototype. Accordingly, reports that have investigated the effect of spatial attention showed that stimuli presented at attended locations elicit much larger P100 components than those presented at unattended locations (Mangun 1995; Mangun et al. 1998). Thus, when faces are upright, P100 might be affected by higher level processing such as attention, facial emotion, and species, rather than by simple low-level visual features. In contrast, inverted faces elicited P100 amplitudes that conformed to a U-shaped function along the face continuum (Fig.5A and B), meaning that greater responses were observed for less ambiguous faces when they were inverted. Thus, P100 amplitude appears to have been modulated by face orientation, which should involve different mechanisms. This suggests that the ambiguous faces in the inverted condition were processed as objects rather than as faces. Overall, the modulation of P100 indicated that it was sensitive to species-specific initial face processing in upright faces.

### N170 is involved in own-species selectivity

N170 and P100 latencies were modulated differently when participants viewed upright faces with equal SF. N170 latency was more prolonged when faces were more simian. Additionally, N170 latency changed in a step-like fashion (Fig.6A) rather than a monotonic-like one. We hypothesized that N170 latency would be more affected by species-specific processing than would P100 latency, such that the N170 would represent a flexion point for discriminating humans from other species. Therefore, the N170 latency may be useful as a marker of categorical boundaries for species discrimination. N170 latencies for inverted faces were longer than those for upright faces, for both human and monkey faces. This is consistent with previous findings (Itier et al. 2006, 2011). Specifically, the generally delayed latency induced by inverted faces in this study reflects a disruption induced by processing non-canonical views of an object (Itier et al. 2006).

The larger N170 amplitude for human (H9) faces than for monkey (H1) faces was consistent with previous findings (de Haan et al. 2002; Itier et al. 2006, 2011). One study comparing N170 components elicited by human and animal faces suggested that the eyes are the most important facial feature for species sensitivity (Itier et al. 2011), and that eyes modulate species-specific face processing. Because we changed the face stimuli, including eyes, with our morphing method, the systematically morphed eyes might have caused the monotonic-like modulation of N170.

It is well known that inverted faces elicit larger N170 components than upright faces (Rossion et al. 1999b). Interestingly, we found that N170 amplitude gradually decreased as the humanness of the face decreased in the inverted face condition; the difference in N170 amplitude between H9 and H1 was evident. However, such modulation was not observed in the upright face condition. This is consistent with the finding that inverted monkey faces have a different modulatory effect than inverted human faces (Itier et al. 2006). Specifically, sensitivity to species is stronger for inverted faces than for upright ones. Processing inverted faces may recruit additional brain regions, such as eye-, object-, or face-selective neurons (Itier et al. 2007; Sadeh and Yovel 2010). Although there is no direct evidence for this, we suggest that the monotonic-like modulation in the inverted face condition was associated with the systematically morphed eyes, because the eyes are an important feature for both species processing and the face-inversion effect (Itier et al. 2007; Rossion 2008). Furthermore, several studies have reported that N170 amplitude is greater for isolated eyes compared with whole faces (Bentin et al. 1996; Itier et al. 2006, 2007).

The response accuracy of judging a face as human could be related to the flexion point of species identification. We obtained a flexion point only in N170 latency. The flexion points were observed using H5 with an upright face and H3 with an inverted face (Fig.6A). The response accuracy of judging a face as human for the upright H5 face was 46.1% and for the inverted H3 face it was 5.5% (Fig.2A). Both behavioral and ERP results indicated that we can almost always correctly categorize species based on upright faces. In contrast, we have difficulty categorizing species using inverted faces, and this was seen in both the behavioral data and N170 latency in terms of shifting the flexion point from the center. This difference in flexion point between upright and inverted faces appears to indicate that we initially process inverted faces as objects rather than as faces.

### SF is important for early feature-based processing

The results of the four-way ANOVA showed that SF significantly modulated all ERP components. Although the degree of amplitude modulation was smaller in the SF controlled condition than in the unequal condition, the way it was modulated was similar for the early components (P100 and N170). The face stimuli used in Experiment 2 (Fig.1B) were much clearer than those used in Experiment 1 (Fig.1C) because SF was not equal. We propose that distinct faces that deviate from a prototype might be easier to categorize than those with equal SF, and that this easiness might cause the lower amplitudes that we observed.

When stimuli had equal SF, P100, and N170 latencies significantly increased (P100 latencies for inverted face was not significant) as the faces appeared less human, and N170 latencies showed a flexion point. However, this was not the case in Experiment 2 when SFs were not equal, indicating that SF affected the latencies. Studies indicate a controversy regarding the effect of face inversion on P100 latencies. Some researchers have reported that inverted faces elicit longer latencies than upright ones (Linkenkaer-Hansen et al. 1998; Itier and Taylor 2004; Itier et al. 2006), while others have stated that orientation has no effect (Rossion et al. 1999a). That we found an effect of SF content on early component latencies confirms our working hypothesis: controlling the low-level visual features of stimuli, including the SF is important for observing the species-specific effect on P100 and the species-boundary effect on N170.

Monotonic-like neurons in the IT area of monkeys appear to reflect changes in the physical properties of face stimuli based on morph level (Sigala et al. 2011). However, P100 and N170 components did not exhibit abrupt changes at intermediate morph levels near the category boundary. Thus, perhaps these components are not directly linked to species categorization, but instead reflect the detection of facial “features” (Bentin and Deouell 2000; Eimer 2000a). Our study suggests that P100 is sensitive to species, which indicates that encoding of human faces may be initiated around 100 msec, and continue during the N170 period. The observed monotonic- or step-like modulations of the early components (P100 and N170) indicate that they are not directly linked to species categorization. Thus, we speculate that early face processing related to species differences might be associated with feature-based processing, such as that of eyes.

### The effect of task requirement on N170

A four-way ANOVA between Experiment 1 and Experiment 3 demonstrated a significant main effect of task requirement. This was probably because N170 amplitudes during the categorization task were much greater than those of the simple reaction task (Fig.6A and C). This difference may result from an attentional effect. In the simple reaction task, we instructed participants to press the button immediately when the face stimulus was perceived. Thus, the time period for spatial attention was very short (about 200–300 msec) compared with that during categorization. Spatial attention can also modulate the structural encoding of faces, as was the case when N170 amplitude was enhanced when subjects attended faces (Holmes et al. 2003). Therefore, we suspect that the short time period for attention in the reaction task led to the attenuation of N170 amplitude.

### The LP component is engaged in species categorization

The mean amplitudes of the LP component in the categorization task were lower in response to highly morphed stimuli than they were to those that were virtually nonmorphed (Fig.7A). In contrast, in the simple reaction task they appear unchanged regardless of the degree of morphing (Fig.7B). These results indicate that the LP component is related to task condition rather than morph type per se, and therefore it might be related to the act of categorizing itself, regardless of the stimuli.

The RTs in the categorization task fitted an inverted U-shaped curve, resembling the behavior of the LP component in terms of the difference between clearly identifiable and ambiguous faces: the LP component was smaller when judging the species was difficult, and the RTs were longer. Thus, the LP component might reflect cognitive processes involved in resolving ambiguity, face identification, and decision-making. As mentioned in the introduction section, a previous ERP study using a categorization task indicated that the LP component (350–450 msec) might reflect decision-making processes and behavioral judgment (Philiastides and Sajda 2006). The LP we observed appears to be similar to these components regarding latency and topography, and could share the same underlying mechanism. An EEG-fMRI integrated study reported that perceptual decision-making about faces produced activation in the fusiform gyrus as well as a wide range of cortical areas, including the lateral occipital complex and the right ventrolateral prefrontal cortex (Philiastides and Sajda 2007). Therefore, we suggest that the LP component reflects activation of a number of cortical regions related to processing complex decisions/identification tasks.

## Conclusions

Our results suggest that face processing regarding species differentiation is reflected by ERP components starting around 100 msec in the occipital region and continuing to around 170 msec in the occipitotemporal region. Species categorization with respect to face identification appears to occur around 400 msec after seeing a face. Moreover, SF control is important for observing modulation of early ERP components by species-specific stimuli.

## Conflict of Interest

We certify that there is no conflict of interest with any financial organization regarding the material discussed in the manuscript.
